# Real-world assessment of the impact of “OFF” episodes on health-related quality of life among patients with Parkinson’s disease in the United States

**DOI:** 10.1186/s12883-021-02074-2

**Published:** 2021-01-30

**Authors:** Andrew Thach, Eddie Jones, Eric Pappert, James Pike, Jack Wright, Alexander Gillespie

**Affiliations:** 1 Sunovion Pharmaceuticals Inc., 84 Waterford Dr, Marlborough, MA 01752 USA; 2 Adelphi Real World, Manchester, UK

**Keywords:** EQ-5D, Health-related quality of life, “OFF” episodes, Parkinson’s disease, PDQ-39, Real-world data

## Abstract

**Background:**

Many patients with Parkinson’s disease (PD) who receive carbidopa/levodopa experience symptom reemergence or worsening, or “OFF” episodes. This study assessed the association of “OFF” episodes with health-related quality of life (HRQoL).

**Methods:**

US-specific data from the 2017 and 2019 Adelphi Real World Disease Specific Programme for PD, a real-world cross-sectional survey, were used. Neurologists provided data for 10–12 consecutive patients with PD who completed the 39-item Parkinson’s Disease Questionnaire (PDQ-39) and the EuroQol 5-Dimension (EQ-5D). Data were grouped by patients who experienced “OFF” episodes versus those who did not and by average hours of daily “OFF” time. Differences between patient groups were assessed for demographics and clinical characteristics; regression analyses were used to model the relationship between HRQoL and “OFF” episodes with age, sex, body mass index, current PD stage on the Hoehn and Yahr scale, and number of concomitant conditions related and unrelated to mobility as covariates.

**Results:**

Data from 722 patients were analyzed. Overall, 321 patients (44%) had “OFF” episodes (mean of 2.9 h of daily “OFF” time). Patients who experienced “OFF” episodes were less likely to work full-time and more likely to live with family members other than their spouse/partner or reside in a long-term care facility than those without “OFF” episodes. The presence of “OFF” episodes, regardless of the average hours of daily “OFF” time, was significantly associated with high scores (reflecting poor HRQoL) on most PDQ-39 dimensions and the summary index and low scores (reflecting poor health status) on the EQ-5D health utility index, visual analog scale (VAS), and all dimensions. Furthermore, increased average hours of daily “OFF” time was significantly correlated with higher scores for all PDQ-39 dimensions and the summary index, as well as with the EQ-5D health utility index and VAS scores. Patients with “OFF” episodes experienced reduced HRQoL even after correcting for potentially confounding variables.

**Conclusions:**

This study demonstrated that the occurrence of “OFF” episodes in patients with PD is associated with reduced HRQoL and that the impact on HRQoL increased incrementally with increasing average hours of daily “OFF” time.

**Supplementary Information:**

The online version contains supplementary material available at 10.1186/s12883-021-02074-2.

## Background

Parkinson’s disease (PD) is the second most common neurodegenerative disorder in the United States and worldwide; only Alzheimer’s disease has a higher prevalence [1–3]. Approximately 1 million people in the United States are estimated to have PD in 2020 [1], with this number forecasted to rise to 1.2 million by 2030 [4]. Symptoms of PD include motor abnormalities such as bradykinesia, rigidity, tremor at rest, and gait and balance problems [5]; however, PD may also be complicated by nonmotor symptoms, such as anxiety and depression [6, 7], cognitive impairment [7–9], constipation, urinary urgency and frequency, dizziness [10], sleep disturbance [11], and psychosis [12].

PD has been shown to adversely affect patients’ health-related quality of life (HRQoL), with the level of impairment related to disease severity, and the impact on physical and social functioning has been most pronounced [13–16]. A Veterans Health Administration study involving ~ 15,000 respondents with PD found that patients had greater impairment on the physical dimension of HRQoL, assessed via the 36-item Short Form Health Survey, than patients with depression, congestive heart failure, stroke, chronic low back pain, arthritis, diabetes, and angina/coronary heart disease, along with greater impairment on the mental health dimension than patients with any of these diseases except depression [17]. In particular, the nonmotor symptoms of PD, such as depression [18], dementia [19], and psychosis [12], have been found to have a substantial impact on HRQoL [20, 21].

Levodopa, in combination with the DOPA decarboxylase inhibitor, carbidopa (ie, carbidopa/levodopa), is the gold-standard treatment for PD and is known to be effective in most patients [22, 23]. However, most patients who receive carbidopa/levodopa develop motor complications, including motor fluctuations and dyskinesias [24]. Motor fluctuations consist of periods when symptoms improve as a result of the beneficial effect of a carbidopa/levodopa dose (“ON”) and periods when symptoms reemerge or worsen (“OFF” episodes) [25]. Motor fluctuations have been reported to occur in 38 to 50% of patients with PD within 2 years of initiating carbidopa/levodopa [26–29] and in nearly 100% of patients after 10 years of carbidopa/levodopa treatment [30]. In a survey conducted by the Michael J. Fox Foundation for Parkinson’s Research, ~ 65% of respondents reported spending at least 2 h of their day in “OFF” time and > 20% reported 4 h or more of “OFF” time [31].

There have been several reports linking the presence of motor fluctuations with further detriments in HRQoL beyond those generally seen in patients with PD [32–34]; however, none of these studies explored the impact of the duration of “OFF” episodes on HRQoL. Using real-world data from patients with PD, the current study assesses differences in HRQoL between patients with PD and “OFF” episodes versus those without “OFF” episodes and investigates the association of the average hours of daily “OFF” time with HRQoL impairment.

## Methods

### Data collection

Data collected from the US-specific Adelphi Real World Disease Specific Programme (DSP) for PD were used in this analysis. The DSP is a real-world, cross-sectional survey of physicians and their consulting patients; details of DSP methodology have been published previously [35]. Surveys were conducted from May to August 2017, and from August to November 2019, in full accordance with the US Health Insurance Portability and Accountability Act of 1996. All data were collected following procedures with ethics committee approval, including obtaining patients’ informed consent.

Neurologists from the United States were identified from published physician registries and invited to participate in the DSP if they met the following eligibility criteria: had initially fulfilled licensure requirements between 1982 and 2015; were responsible for treatment decisions for patients with PD; and saw at least 10 patients with PD in a typical week.

Neurologists completed a patient record form (PRF) for 10 to 12 consecutive adult patients with PD. Patient history was obtained retrospectively through review of the patient’s complete medical records held at the neurologist’s office. Patients were also invited to complete a patient self-completion form, which recorded information about how their PD had impacted their lives, with specific measures included to capture HRQoL and health status.

### Measures and variables

Information recorded in the PRF included demographics, clinical characteristics, personal circumstances (employment status and living situation), the patient’s current PD stage on the Hoehn and Yahr scale [36], current medication and medication history, and any concomitant conditions. Specific questions on the PRF captured whether patients experienced “OFF” episodes and, if so, the average hours of daily “OFF” time.

The patient self-completion form included 2 patient-reported measures: the 39-item Parkinson’s Disease Questionnaire (PDQ-39) and the EuroQol 5-Dimension (EQ-5D). The PDQ-39 assesses difficulties experienced by patients with PD across 8 dimensions (mobility, activities of daily living, emotional well-being, stigma, social support, cognitions, communication, and bodily discomfort) and provides a summary index [37]. Each of the 39 items has 5 response options, which are scored between 0 (never) and 4 (always); these are used to calculate dimension scores ranging from 0 (never have any difficulty) to 100 (always have difficulty). The summary index is the mean of the 8 dimension scores. The EQ-5D consists of 5 dimensions (mobility, self-care, usual activities, pain/discomfort, and anxiety/depression), each with response options indicating no problems, moderate problems, or extreme problems, and a 20-cm visual analog scale (VAS) describing the respondent’s general health status at the time of completion [38]. Application of country-specific scoring algorithms to the 5 dimension scores of the EQ-5D results in a single health utility index score, with 1 indicating perfect health, and 0 or below indicating that the patient’s health would be regarded, from a societal perspective, as being in a state equal to or worse than death [39, 40].

### Analysis

To be included in this analysis, patients must have had a neurologist-confirmed diagnosis of PD, were not participating in another clinical trial, were receiving carbidopa/levodopa at the time of the survey, and had valid data available for “OFF” episodes (recorded by the neurologist as being present along with average hours of daily “OFF” time or absent and experiencing 0 h) and all outcomes and covariates included in the regression analyses described below. While it was not specifically recorded, patients who required a legal guardian or significant assistance may have been captured in the study population.

Demographic data, personal circumstances, and clinical characteristics were analyzed descriptively, and the statistical significance of differences between patients who experienced “OFF” episodes and those who did not were assessed using the Student’s *t* test for continuous variables, χ^2^ test for multi-categorical variables, and Fisher’s exact test for binary variables. The association of average hours of daily “OFF” time with demographics, personal circumstances, and clinical characteristics was analyzed by comparison of patients who experienced 0 h of “OFF” time vs 1 h, 2 h, 3 h, and ≥ 4 h of “OFF” time. No adjustment was made for multiple testing.

Regression analyses were used to model the relationship between HRQoL and the presence of “OFF” episodes and the average hours of daily “OFF” time. Linear regressions, providing effect coefficients, were performed with the PDQ-39 summary index and dimensions and the EQ-5D health utility index and VAS scores as dependent variables. Odds ratios were calculated from ordered logistic regressions for each PDQ-39 item and EQ-5D dimension, allowing the item/dimension that showed the greatest discrimination between patients with and without “OFF” episodes to be identified. The occurrence of “OFF” episodes was the main independent variable of interest, with analyses controlling for both the presence/absence of any “OFF” episodes and for the average hours of daily “OFF” time. Regression analyses were adjusted for other independent variables (age, sex, body mass index, and the number of concomitant conditions related or unrelated to mobility). Time since PD diagnosis was not included as an independent variable, as this was significantly correlated with both age and Hoehn and Yahr stage but was not completed for a substantial proportion of patients.

Standard errors were adjusted, using the Huber and White sandwich estimator of variance or the robust estimator of variance [41], to allow for intragroup correlation within each reporting neurologist, relaxing the usual requirement that the observations be independent. Adjusted predictions were produced for each regression, i.e., the predicted outcome for an outcome measure was produced for each hour of daily “OFF” time, assuming sample average values for other regression covariates.

All analyses were conducted in Stata v15.1 (StataCorp LLC, College Station, Texas, USA) [42].

## Results

### Patient demographics, personal circumstances, clinical characteristics, and current treatment

A total of 130 neurologists provided data for 722 patients who were receiving carbidopa/levodopa and had all data available required for the analyses. Patients ranged in age from 26 to 90 years, and most were male (62%), retired (58%), and living with their spouse or partner (80%; Table 1). Slightly over half of the patients (55%) had a Hoehn and Yahr score of < 3, indicating mild PD, and only 13% had a Hoehn and Yahr score of 4 or 5, indicative of PD symptoms resulting in significant disability. Mean age at diagnosis was just over 60 years, and the mean duration of PD was slightly over 4 years but ranged to over 20 years (Table 1). In addition to carbidopa/levodopa, patients were also prescribed antiparkinsonian treatments from other pharmacologic classes, including dopamine agonists (25%), catechol-O-methyl transferase inhibitors (17%), monoamine oxidase type B inhibitors (16%), and N-methyl-D-aspartate antagonists (13%; Table 1).

**Table 1:** Patient demographics, personal circumstances, clinical characteristics, and current treatment

	Overall (***N*** = 722)	No “OFF” episodes (***n*** = 401)	“OFF” episodes (***n*** = 321)	***P*** value
**Age, y**^a^				
*N*	722	401	321	0.253^b^
Mean (SD)	67.3 (11.6)	67.7 (10.5)	66.7 (12.7)	
Min, max	26, 90	26, 90	37, 90	
**Sex**				
*N*	722	401	321	0.202^c^
Male	447 (61.9)	240 (59.9)	207 (64.5)	
Female	275 (38.1)	161 (40.1)	114 (35.5)	
**Employment status**				
*N*	711	394	317	0.008^c^
Works full-time	118 (16.6)	78 (19.8)	40 (12.6)	
Works part-time	90 (12.7)	38 (9.6)	52 (16.4)	
Long-term sick leave	4 (0.6)	2 (0.5)	2 (0.6)	
Homemaker	64 (9.0)	42 (10.7)	22 (6.9)	
Student	1 (0.1)	1 (0.3)	0	
Retired	410 (57.7)	221 (56.1)	189 (59.6)	
Unemployed	24 (3.4)	12 (3.0)	12 (3.8)	
**Long-term sick leave/retired/unemployed due to PD**				
*N*	387	212	175	< 0.001^c^
Yes	70 (18.1)	27 (12.7)	43 (24.6)	
No	317 (81.9)	185 (87.3)	132 (75.4)	
**Living situation**				
*N*	708	394	314	0.001^c^
Alone	63 (8.9)	46 (11.7)	17 (5.4)	
With spouse/partner	568 (80.2)	318 (80.7)	250 (79.6)	
With other family	58 (8.2)	22 (5.6)	36 (11.5)	
With friends	1 (0.1)	1 (0.3)	0	
Long-term care facility	14 (2.0)	5 (1.3)	9 (2.9)	
Sheltered housing	1 (0.1)	1 (0.3)	0	
Other	3 (0.4)	1 (0.3)	2 (0.6)	
**Current Hoehn and Yahr score**				
*N*	722	401	321	< 0.001^c^
1	59 (8.2)	54 (13.5)	5 (1.6)	
1.5	87 (12.0)	73 (18.2)	14 (4.4)	
2	101 (14.0)	73 (18.2)	28 (8.7)	
2.5	152 (21.1)	74 (18.5)	78 (24.3)	
3	231 (32.0)	104 (25.9)	127 (39.6)	
4	83 (11.5)	21 (5.2)	62 (19.3)	
5	9 (1.2)	2 (0.5)	7 (2.2)	
**Age at PD diagnosis, y**				
*N*	555	319	236	< 0.001^b^
Mean (SD)	61.2 (11.4)	62.9 (10.4)	58.9 (12.3)	
Min, max	24.6, 88.0	24.6, 88.0	29.8, 84.3	
**Time since PD diagnosis, y**				
*N*	555	319	236	< 0.001^b^
Mean (SD)	4.5 (3.6)	3.6 (3.1)	5.9 (3.9)	
Min, max	0.0, 20.9	0.0, 17.6	0.0, 20.9	
**Current prescribed treatment classes**				
*N*	722	401	321	
Carbidopa/levodopa	722 (100.0)	401 (100.0)	321 (100.0)	1.000^c^
COMT inhibitor	119 (16.5)	48 (12.0)	71 (22.1)	< 0.001^c^
Dopamine agonist	177 (24.5)	73 (18.2)	104 (32.4)	< 0.001^c^
MAO-B inhibitor	118 (16.3)	50 (12.5)	68 (21.2)	0.002^c^
NMDA receptor antagonist	90 (12.5)	24 (6.0)	66 (20.6)	< 0.001^c^
Device-aided treatment	37 (5.1)	11 (2.7)	26 (8.1)	0.001^c^
Other	20 (2.8)	7 (1.7)	13 (4.0)	0.061^c^
**Number of prescribed treatment classes**				
*N*	722	401	321	
Mean (SD)	1.8 (0.9)	1.5 (0.7)	2.1 (1.1)	< 0.001^b^
Min, Max	1.0, 7.0	1.0, 4.0	1.0, 7.0	
**Levodopa equivalent daily dose**				
*N*	698	385	313	< 0.001^c^
1–499 mg	468 (67.0)	282 (73.2)	186 (59.4)	
500–999 mg	187 (26.8)	84 (21.8)	103 (32.9)	
1000+ mg	43 (6.2)	19 (4.9)	24 (7.7)	

Data are reported as *n* (%) unless specified otherwise

^a^Patients reported to be aged ≥90 years of age were assumed to be 90 years of age in this analysis. ^b^*P* values were calculated by t-tests. ^c^*P* values were calculated by Fisher’s exact/χ^2^ tests

*COMT* catechol-O-methyl transferase; *MAOB* monoamine oxidase type B; *NMDA* N-methyl-D-aspartate; *PD* Parkinson’s disease; *SD* standard deviation

### Relationship of “OFF” episodes with demographic data, personal circumstances, clinical characteristics, and current treatment

**Fig. 1:**
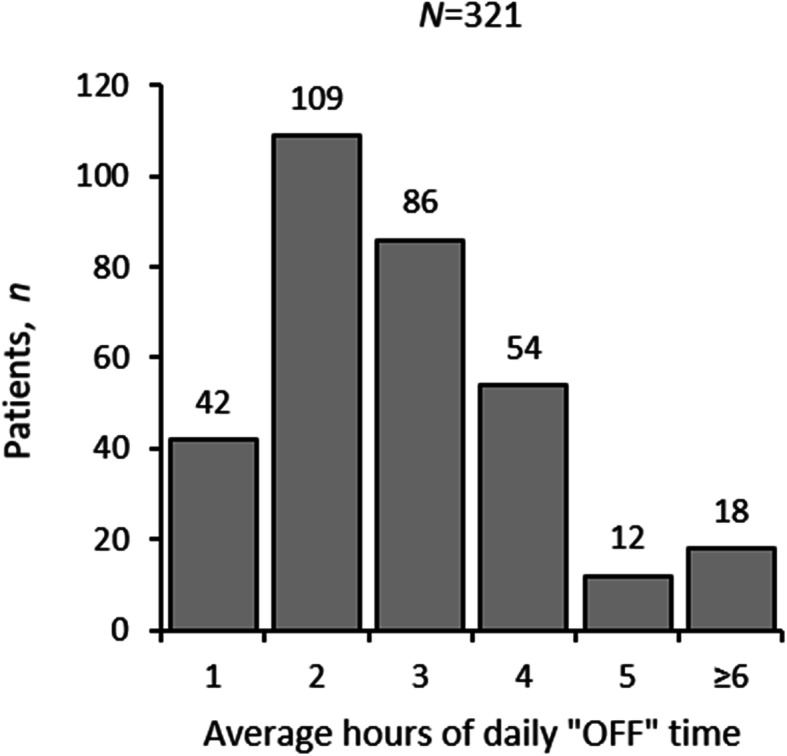
Average hours of daily “OFF” time experienced

Of the 722 patients, 321 (44%) experienced an average of at least 1 h of daily “OFF” time (Fig. 1). The vast majority (94%) of patients with “OFF” episodes experienced 1 to 5 h of daily “OFF” time, with 2 h per day being the most common duration (mean [standard deviation], 2.9 [1.5] hours per day). Eighteen patients experienced 6 or more hours of daily “OFF” time.

No relationship between age or sex and the presence or absence of “OFF” episodes was observed (Table 1). There was a significant association between employment status and the occurrence of “OFF” episodes (*P* = 0.008); patients who experienced “OFF” episodes were less likely to be in full-time employment and more likely to be in part-time employment or retired. In addition, patients with “OFF” episodes more frequently reported that they were on long-term sick leave, retired, or unemployed due to their PD than patients without “OFF” episodes (*P* <  0.001). There was a significant association between the occurrence of “OFF” episodes and patients’ living situation (*P* = 0.001); a higher proportion of patients without “OFF” episodes lived alone, while higher proportions of patients with “OFF” episodes lived with family members other than their spouse/partner or in a long-term care facility. Patients who experienced “OFF” episodes were younger at diagnosis and had PD for a longer duration than those who did not experience “OFF” episodes (both *P* <  0.001; Table 1). The mean number of prescribed treatment classes and levodopa equivalent daily dose was significantly associated with the presence of “OFF” episodes (both *P* <  0.001; Table 1).

### Health-related quality of life

#### Occurrence of “OFF” episodes

Poorer HRQoL was associated with the presence of “OFF” episodes. Scores for the PDQ-39 summary index (*P* <  0.001) and all PDQ-39 dimensions (*P* <  0.05) except for stigma, social support, and bodily discomfort were significantly associated with the presence or absence of “OFF” episodes (Table 2).

**Table 2:** Relationship between PDQ-39 scores and presence/absence of “OFF” episodes (*N* = 722)

PDQ-39 dimensions	Coefficient (95% CI)^a^	***P*** value
Mobility	+ 7.9 (4.4, 11.4)	< 0.001
Activities of daily living	+ 6.7 (3.2, 10.1)	< 0.001
Emotional well-being	+ 4.5 (0.7, 8.3)	0.019
Stigma	+ 3.0 (−0.1, 6.1)	0.057
Social support	+ 2.3 (−0.7, 5.3)	0.134
Cognitions	+ 6.5 (3.5, 9.4)	< 0.001
Communication	+ 9.0 (6.0, 11.9)	< 0.001
Bodily discomfort	+ 1.7 (−3.0, 6.5)	0.470
Summary index	+ 5.2 (2.8, 7.6)	< 0.001

^a^Effect coefficient for difference between patients with/without “OFF” episodes from linear regression; + indicates a higher score on the PDQ-39 dimension or summary index, indicating poorer health-related quality of life, in patients with “OFF” episodes compared with patients without “OFF” episodes

*CI* confidence interval; *PDQ-39* 39-item Parkinson’s Disease Questionnaire

Significantly higher scores, indicating poorer HRQoL, were reported by patients with “OFF” episodes versus those without “OFF” episodes for most items on the PDQ-39 (Table 3). For the mobility dimension, the “Been confined to the house more than you would like” item showed the greatest difference between patients with and without “OFF” episodes. In the activities of daily living dimension, the “Had problems writing clearly” item showed the greatest association with “OFF” episodes. High scores on the “Felt isolated and lonely” item showed the greatest association with “OFF” episodes across all items in the emotional well-being dimension. All items comprising the stigma dimension had higher scores in the presence of “OFF” episodes, but no statistical difference was seen compared with the absence of “OFF” episodes except for the “Avoided situations which involve eating or drinking in public” item. For the social support dimension, higher scores were reported on all items for patients with “OFF” episodes, but differences were nonsignificant compared with patients without “OFF” episodes except for the “Had problems with your close personal relationships” item. High scores for the “Had problems with your concentration” item showed the greatest association with “OFF” episodes in the cognitions dimension. For the communication dimension, the “Had difficulty with your speech” item showed the greatest difference between patients with and without “OFF” episodes. Higher scores were reported for all items in the bodily discomfort dimension by patients with “OFF” episodes compared with those without “OFF” episodes, but these differences were nonsignificant.

**Table 3:** Relationship between PDQ-39 items and presence/absence of “OFF” episodes (*N* = 722)

PDQ-39 items	Odds ratio (95% CI)^a^	***P*** value
**Mobility**		
Had difficulty doing the leisure activities which you would like to do	2.2 (1.5, 3.0)	< 0.001
Had difficulty looking after your home	1.9 (1.4, 2.6)	< 0.001
Had difficulty carrying bags of shopping	1.7 (1.2, 2.3)	0.002
Had problems walking half a mile	1.4 (1.0, 2.0)	0.028
Had problems walking 100 yards	2.0 (1.5, 2.7)	< 0.001
Had problems getting around the house as easily as you would like	2.2 (1.6, 3.0)	< 0.001
Had difficulty getting around in public	1.8 (1.3, 2.6)	< 0.001
Needed someone else to accompany you when you went out	1.9 (1.3, 2.6)	0.001
Felt frightened or worried about falling over in public	1.5 (1.0, 2.1)	0.040
Been confined to the house more than you would like	2.5 (1.9, 3.3)	< 0.001
**Activities of daily living**		
Had difficulty washing yourself	1.8 (1.3, 2.4)	< 0.001
Had difficulty dressing yourself	1.8 (1.3, 2.4)	< 0.001
Had problems doing up buttons or shoelaces	1.7 (1.3, 2.3)	0.001
Had problems writing clearly	2.2 (1.6, 2.9)	< 0.001
Had problems cutting up your food	1.4 (1.0, 2.0)	0.027
Had difficulty holding a drink without spilling it	1.3 (0.9, 1.8)	0.108
**Emotional well-being**		
Felt depressed	1.3 (0.9, 1.9)	0.176
Felt isolated and lonely	1.7 (1.2, 2.3)	0.003
Felt weepy or tearful	1.5 (1.0, 2.1)	0.055
Felt angry or bitter	1.5 (1.1, 2.1)	0.014
Felt anxious	1.5 (1.0, 2.2)	0.026
Felt worried about your future	1.2 (0.8, 1.8)	0.391
**Stigma**		
Felt you had to conceal your Parkinson’s from people	1.3 (0.9, 1.8)	0.127
Avoided situations which involve eating or drinking in public	1.5 (1.1, 2.1)	0.010
Felt embarrassed in public due to having Parkinson’s disease	1.3 (0.9, 1.8)	0.100
Felt worried by other people’s reaction to you	1.2 (0.9, 1.7)	0.246
**Social support**		
Had problems with your close personal relationships	1.5 (1.0, 2.1)	0.048
Lacked support in the ways you need from your spouse or partner	1.2 (0.8, 2.0)	0.399
Lacked support in the ways you need from your family or close friends	1.3 (0.8, 2.0)	0.295
**Cognitions**		
Unexpectedly fallen asleep during the day	1.8 (1.3, 2.5)	< 0.001
Had problems with your concentration	2.1 (1.5, 2.9)	< 0.001
Felt your memory was bad	1.3 (1.0, 1.9)	0.090
Had distressing dreams or hallucinations	1.8 (1.3, 2.5)	< 0.001
**Communication**		
Had difficulty with your speech	3.4 (2.3, 4.9)	< 0.001
Felt unable to communicate with people properly	3.2 (2.3, 4.5)	< 0.001
Felt ignored by people	2.0 (1.4, 2.9)	< 0.001
**Bodily discomfort**		
Had painful muscle cramps or spasms	1.2 (0.8, 1.9)	0.328
Had aches and pains in your joints or body	1.1 (0.7, 1.7)	0.801
Felt unpleasantly hot or cold	1.3 (0.9, 1.9)	0.189

^a^Odds ratio for difference between patients with/without “OFF” episodes from ordered logistic regression; an odds ratio > 1 indicates higher likelihood of problems in a PDQ-39 item, indicating poorer health-related quality of life, in patients with “OFF” episodes compared with patients without “OFF” episodes

*CI* confidence interval; *PDQ-39* 39-item Parkinson’s Disease Questionnaire

Significantly lower EQ-5D scores (indicating poorer health status) were reported by patients who experienced “OFF” episodes than those who did not experience “OFF” episodes for the mobility (*P* = 0.002), usual activities (*P* <  0.001), and anxiety/depression (*P* <  0.001) dimensions (Table 4). Lower EQ-5D health utility index and VAS scores were also significantly associated with the presence of “OFF” episodes (both *P* <  0.001), supporting the finding that “OFF” episodes are associated with a detriment in health status (Table 4).

**Table 4:** Relationship between EQ-5D scores and presence/absence of “OFF” episodes (*N* = 722)

EQ-5D dimensions	Odds ratio (95% CI)^a^	***P*** value
Mobility	2.1 (1.3, 3.5)	0.002
Self-care	1.4 (0.9, 2.1)	0.112
Usual activities	2.6 (1.8, 3.7)	< 0.001
Pain/discomfort	1.2 (0.8, 1.8)	0.467
Anxiety/depression	1.6 (1.1, 2.2)	< 0.001
	**Coefficient (95% CI)**^**b**^	***P*** **value**
Health utility index	−0.04 (−0.07, −0.02)	< 0.001
VAS	−6.75 (−9.49, −4.00)	< 0.001

^a^Odds ratio for difference between patients with/without “OFF” episodes from ordered logistic regression on the categorical EQ-5D responses (indicating no problem/some problem/extreme problem). ^b^Effect coefficient for difference between patients with/without “OFF” episodes from linear regression on EQ-5D utility index and VAS; − indicates a lower score on the EQ-5D utility index or VAS, indicating poorer health status, in patients with “OFF” episodes compared with patients without “OFF” episodes

*CI* confidence interval; *EQ-5D* EuroQol 5-Dimension; *VAS* visual analog scale

#### Average hours of daily “OFF” time

**Fig. 2:**
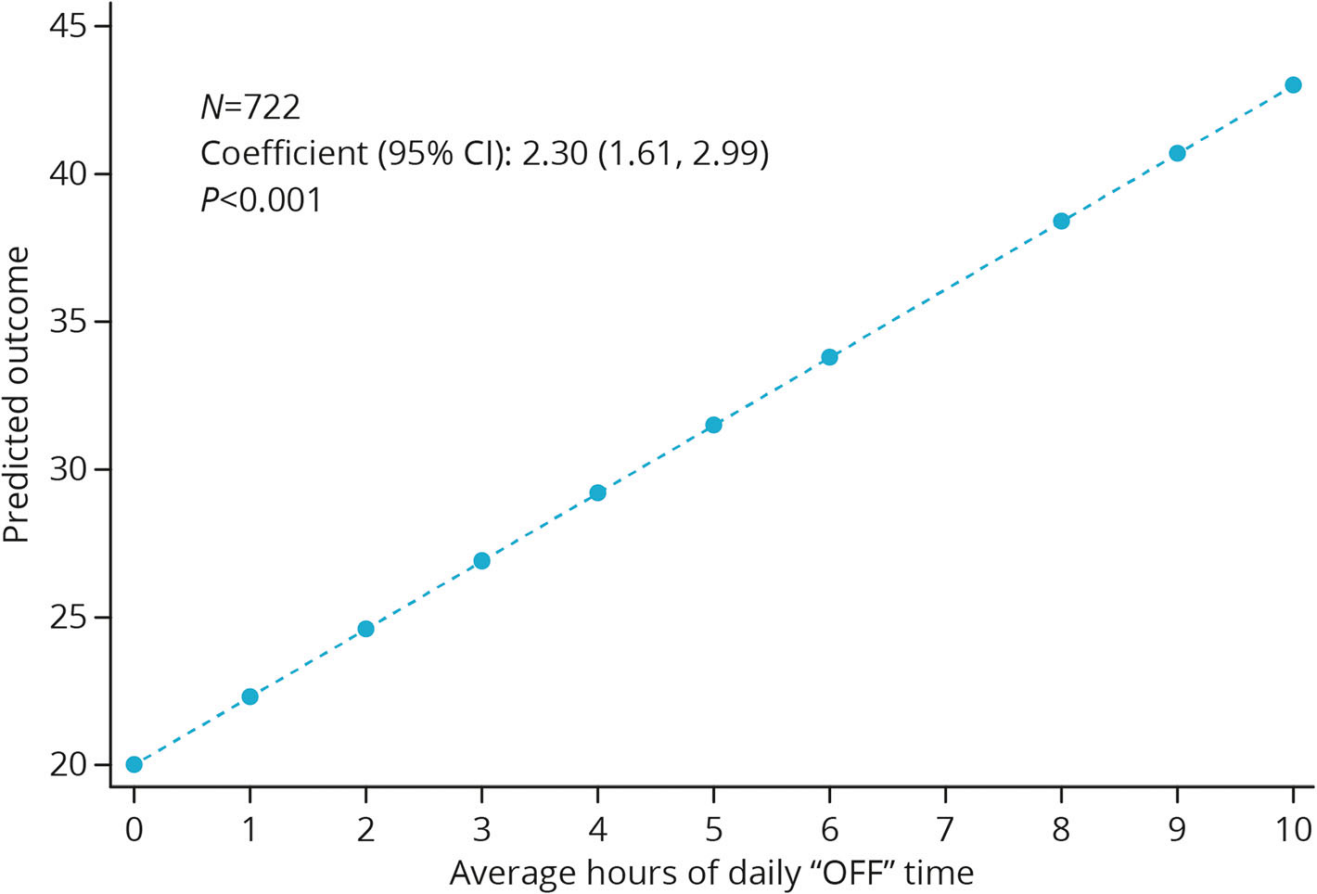
Linear regression analyses of relationship between PDQ-39 summary index and average hours of daily “OFF” time. *CI* confidence interval; *PDQ-39* 39-Item Parkinson’s Disease Questionnaire

Scores for all PDQ-39 dimensions and the summary index were significantly correlated with the average hours of daily “OFF” time, with longer daily duration linked to scores indicative of poorer HRQoL (*P* <  0.05; Fig. 2 and Additional file 1). Linear regression analyses predicted that, for every additional hour of daily “OFF” time, there would be increases of 2.73, 2.60, 2.12, 1.63, 1.04, 2.80, 3.55, and 1.91 on the PDQ-39 mobility, activities of daily living, emotional well-being, stigma, social support, cognitions, communication, and bodily discomfort dimensions, respectively (Additional file 1), together with an increase of 2.30 on the PDQ-39 summary index (Fig. 2).

**Fig. 3:**
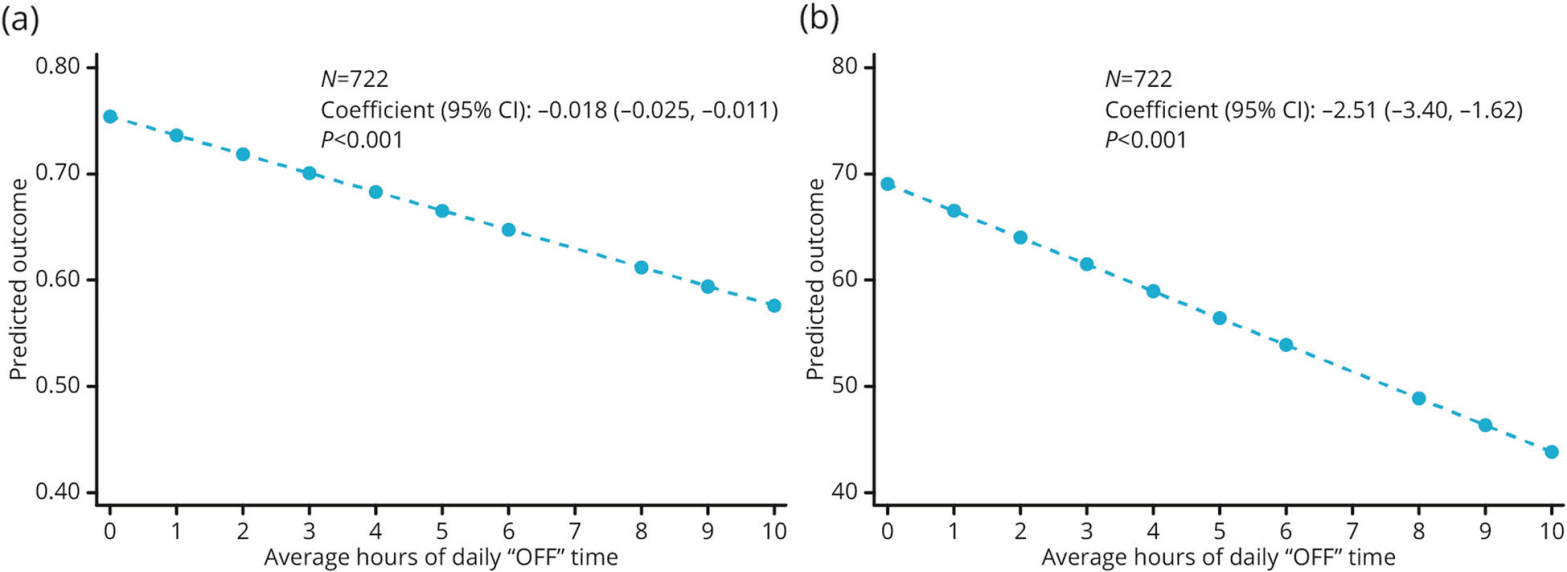
Linear regression analyses of relationship between EQ-5D and average hours of daily “OFF” time. (a) Health utility index and (b) visual analog scale. *CI* confidence interval; *EQ-5D* EuroQol 5-Dimension

Both the EQ-5D health utility index and VAS scores were significantly negatively correlated with the average hours of daily “OFF” time (both *P* <  0.001; Fig. 3). Linear regression analyses predicted that, for every additional hour of daily “OFF” time, there would be a decrease of 0.02 in the EQ-5D utility index and a decrease of 2.5 in the EQ-5D VAS, indicating deteriorating health status with increasing hours of daily “OFF” time (Fig. 3).

## Discussion

Our analysis included real-world data from over 700 patients with PD, nearly half of whom experienced “OFF” episodes. Among those who experienced “OFF” episodes, patients had a mean of 2.9 average hours of daily “OFF” time, and the most commonly reported duration was 2 h per day. The presence of “OFF” episodes was significantly associated with a PD diagnosis at a younger age, longer duration of PD, and a reduced likelihood of working full-time or living alone.

HRQoL was evaluated using the PDQ-39, a disease-specific HRQoL questionnaire widely used in PD [43], and the EQ-5D, a well-established generic health status questionnaire that allows comparison of health status across diseases [38, 44]. Patients who experienced “OFF” episodes had significantly poorer HRQoL (as measured by the PDQ-39) and significantly worse health status (as measured by the EQ-5D) than patients without “OFF” episodes. The finding of reduced HRQoL and poorer health status among patients with “OFF” episodes was seen even when regression analyses were adjusted for potentially confounding variables, such as age and mobility-related comorbidities.

The impact on HRQoL and health status was directly related to the average hours of daily “OFF” time that patients experienced, with significantly correlated scores seen on all dimensions of the PDQ-39, the PDQ-39 summary index, the EQ-5D health utility index, and the EQ-5D VAS.

When interpreting these findings, the minimal clinically important difference (smallest difference that a patient perceives as meaningful) for the instruments used should be considered. The minimal clinically important difference for the PDQ-39 summary has been reported to increase with increasing severity of PD; yet, a study of a large pool of patients with varying severities of PD estimated the minimal clinically important improvement as − 4.72 and minimal clinically important worsening as + 4.22 using both anchor- and distribution-based techniques [45]. In our study, the predictive margin from regression analysis showed a difference of 5.2 in the PDQ-39 summary index between patients who experienced “OFF” episodes and those without “OFF” episodes, suggesting that differences between these groups was clinically important. However, a difference of 0.04 in EQ-5D utility index was observed between patients with and without “OFF” episodes, which is less than the reported minimally important difference of 0.074 [46].

Five of the 8 PDQ-39 dimensions showed a statistically significant difference between patient groups, with no significant difference demonstrated for stigma, social support, or bodily discomfort. However, only 5.4% of patients who experienced “OFF” episodes lived alone, compared with 11.7% of those without “OFF” episodes; thus, family support might have offset the impact of “OFF” episodes on the PDQ-39 stigma and social support dimensions.

Evaluation of the findings for the 8 dimensions of the PDQ-39 indicated that the greatest impact of “OFF” episodes on HRQoL resulted from detriments in communication, mobility, and activities of daily living. Odds ratios for “OFF” episodes versus no “OFF” episodes were ≥ 3.0 for 2 of the 3 PDQ-39 communication-related items and ≥ 2.0 for 4 of the 10 PDQ-39 mobility-related items. Findings from the EQ-5D were similar to those of the PDQ-39, with differences between patients who experienced “OFF” episodes and those who did not being most marked for the EQ-5D mobility and usual activities dimensions. Generally, “OFF” episodes did not appear to have as close of an association with emotional, social, and pain-related aspects of HRQoL as those related to physical functioning.

The finding that “OFF” episodes is associated with reduced physical functioning is expected given the reemergence or worsening of motor symptoms during “OFF” episodes [47]. Several published studies have reported an association between “OFF” episodes and detriments in HRQoL. In a previous study using data from 5 European countries, the presence of “OFF” episodes was shown to be associated with reduced HRQoL, as assessed using the PDQ-39 and EQ-5D [33]. Consistent with our study, an observational study in France found that the mobility, activities of daily living, and communication dimensions of the PDQ-39 all showed a greater impact of PD among patients who experienced “OFF” episodes than for those without “OFF” episodes [32]. The impact of “OFF” episodes on HRQoL as assessed with the PDQ-39 was also reported in a Brazilian study; however, in contrast to our findings, this study showed a negative effect of “OFF” episodes on bodily discomfort and did not show emotional well-being or cognitions to be impacted [34]. Neither of these published studies examined the relationship of the average hours of daily “OFF” time with HRQoL. Therapeutic approaches that reduce “OFF” episodes have been shown to result in improvements in activities of daily living, HRQoL, and nonmotor symptoms [48].

Several methodological limitations should be noted. As PRFs were completed for the next 10 to 12 consecutive patients with PD regardless of whether they were consulting the neurologist during either an initial or follow-up visit, the sample collected was pseudo-random, rather than a truly random sample. This survey was cross-sectional rather than longitudinal; as such, data may be used to assess the association between factors but not to assess causality. Similar to other studies of this type, the methodology relies on accurate reporting by neurologists and patients. As only those patients with sufficient data available to perform the analysis were included in the study, it is possible that the study population was not totally representative of the entire population of patients with PD. Age, which often correlates well with proxies of disease severity such as time since diagnosis, was controlled for in the regression analyses; however, time since PD diagnosis was not, as this was missing for a substantial proportion of patients and including it would have thus reduced the sample size considerably. The analyses also did not control for concomitant dementia or psychosis, which can have a significant impact on HRQoL. Hence, it is possible that differences in HRQoL might reflect the consequences of worsening PD, rather than the specific occurrence of “OFF” episodes. Whilst acknowledging these limitations, a substantial body of data from a large representative population of patients with PD was included in the analysis.

## Conclusions

These findings show that “OFF” episodes in patients with PD are associated with reduced HRQoL, with the impact increasing incrementally with increasing average hours of daily “OFF” time. Further study is warranted to assess the impact of treatments to manage “OFF” episodes on improving patients’ HRQoL.

## Supplementary Information

**Additional file 1: Figure S1**. Linear regression analyses of relationship between PDQ-39 dimensions and average hours of daily “OFF” time. (a) Mobility, (b) Activities of daily living, (c) Emotional well-being, (d) Stigma, (e) Social support, (f) Cognitions, (g) Communication, and (h) Bodily discomfort. CI confidence interval; PDQ-39 39-Item Parkinson’s Disease Questionnaire.

## Data Availability

The datasets used and/or analyzed during the current study are available from the corresponding author on reasonable request.

## Abbreviations

CI: Confidence interval
DSP: Disease Specific Programme
EQ-5D: EuroQol 5-Dimension
HRQoL: Health-related quality of life
PD: Parkinson’s disease
PDQ-39: 39-Item Parkinson’s Disease Questionnaire
PRF: Patient record form
VAS: Visual analog scale
