# Supplementary material for: Real-world assessment of the impact of “OFF” episodes on health-related quality of life among patients with Parkinson’s disease in the United States
Source: BMC Neurol. 2021 Jan 30;21:46. doi: 10.1186/s12883-021-02074-2 (PMC7846980; doi:10.1186/s12883-021-02074-2)

**Additional file 1.**

**Figure S1.** Linear regression analyses of relationship between PDQ-39 dimensions and average hours of daily “OFF” time. (a) Mobility, (b) Activities of daily living, (c) Emotional well-being, (d) Stigma, (e) Social support, (f) Cognitions, (g) Communication, and (h) Bodily discomfort. CI confidence interval; PDQ-39 39-Item Parkinson’s Disease Questionnaire.


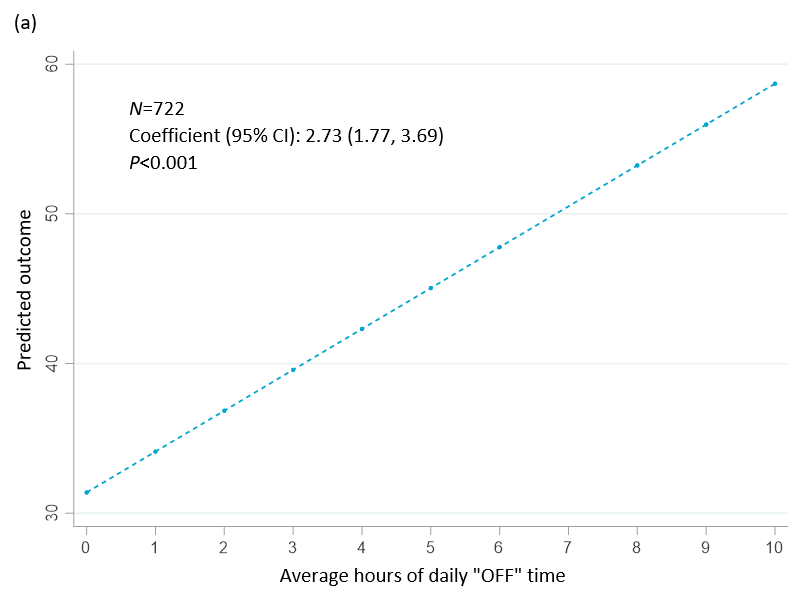


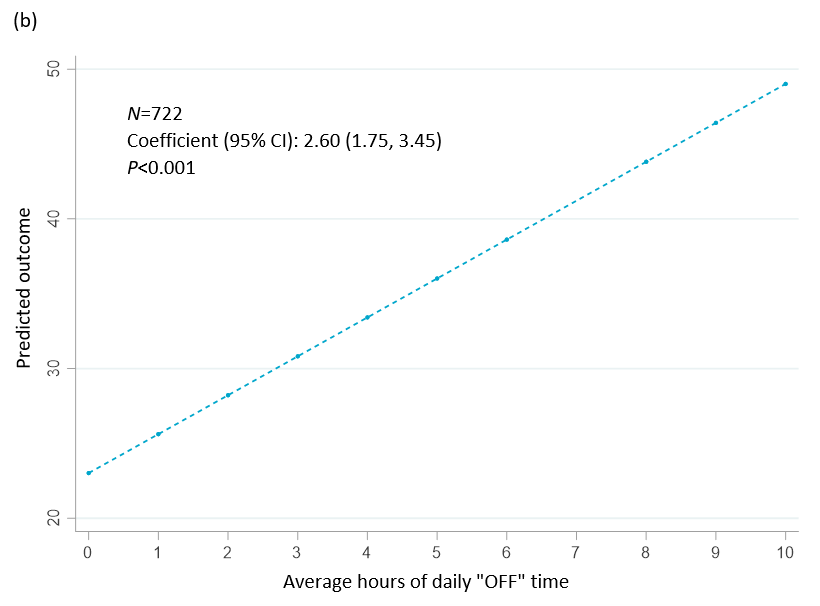


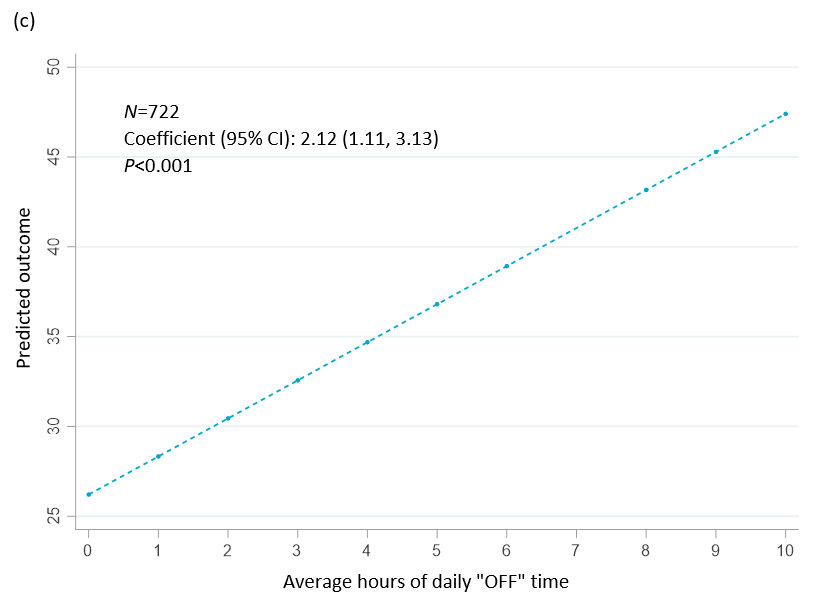


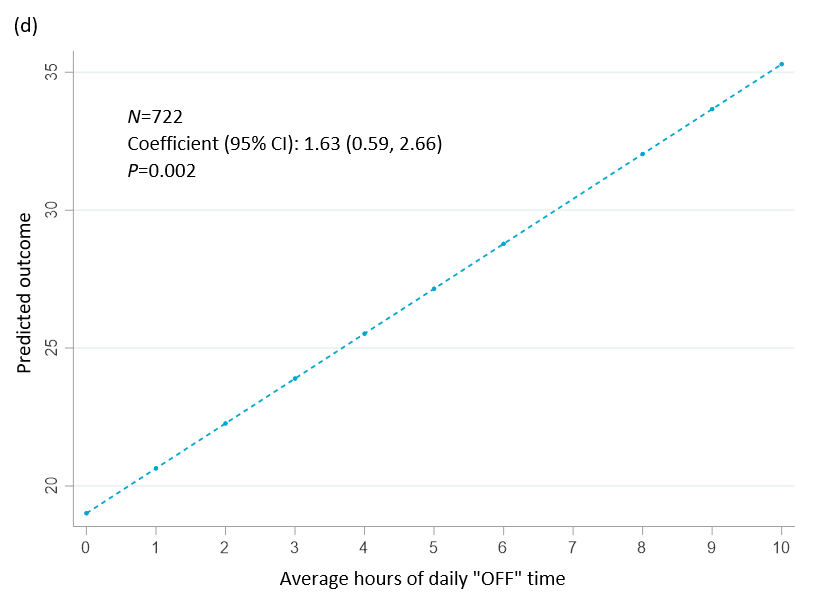


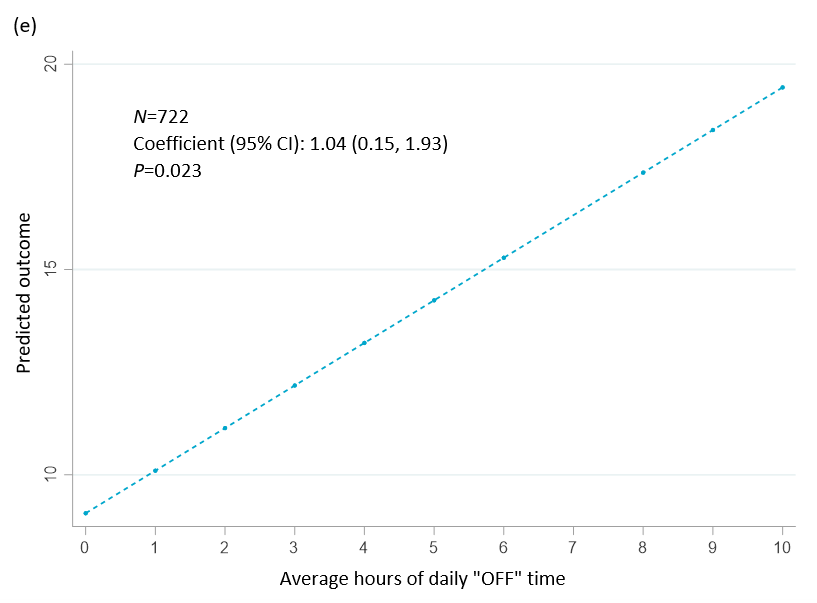


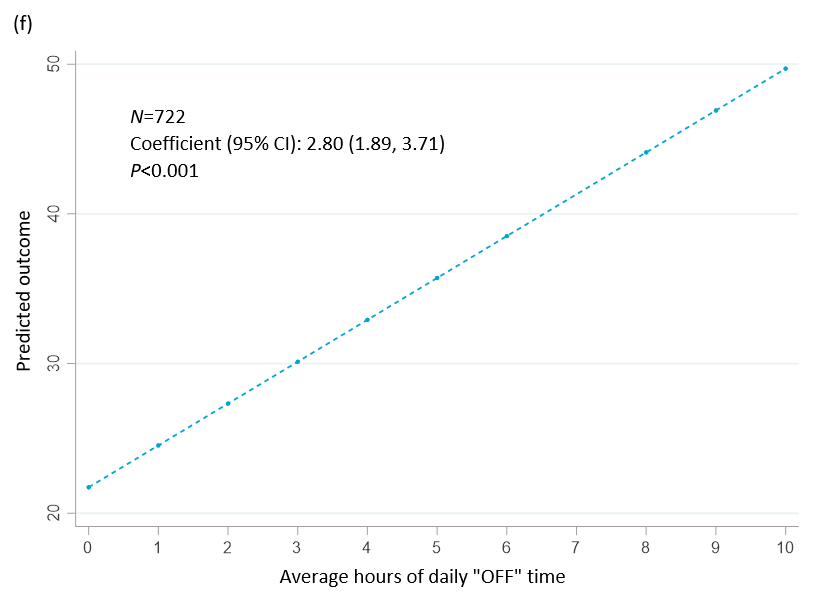


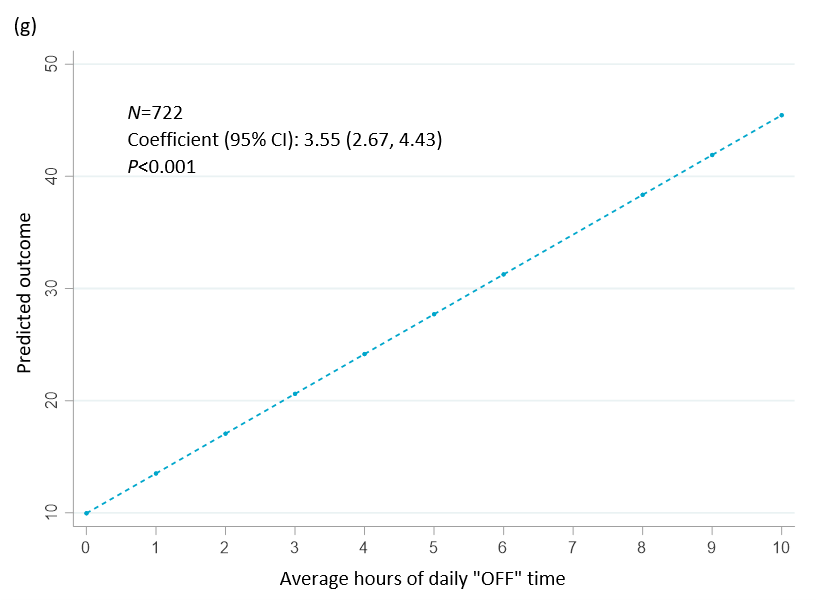


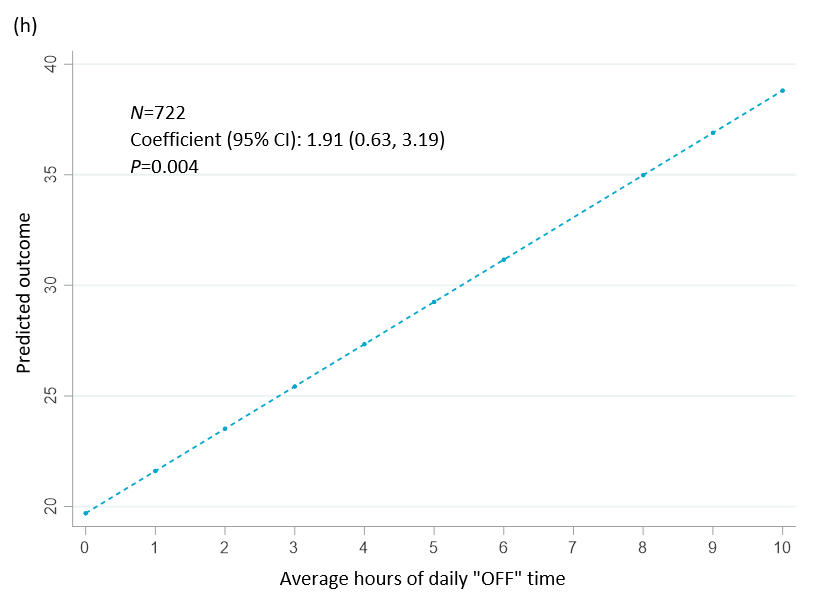

Supplement: Supplementary file 1 — Additional file 1: Figure S1. Linear regression analyses of relationship between PDQ-39 dimensions and average hours of daily “OFF” time. (a) Mobility, (b) Activities of daily living, (c) Emotional well-being, (d) Stigma, (e) Social support, (f) Cognitions, (g) Communication, and (h) Bodily discomfort. CI confidence interval; PDQ-39 39-Item Parkinson’s Disease Questionnaire. [file 12883_2021_2074_MOESM1_ESM.docx]
